# 9-[4,5-Bis(benzyl­sulfan­yl)-1,3-dithiol-2-yl­idene]-4,5-diaza­fluorene

**DOI:** 10.1107/S1600536808018667

**Published:** 2008-06-28

**Authors:** Xu-Dong Zhong, Yu-Lan Zhu, Xue Wu, Jing-Yi Jin, Xue-Ling Tang

**Affiliations:** a Department of Chemistry, College of Science, Yanbian University, Yanji 133002, People’s Republic of China; b Jiangsu Key Laboratory for the Chemistry of Low-Dimensional Materials, Huaiyin Teachers’ College, Huaian 223300, People’s Republic of China

## Abstract

In rhe title compound, C_28_H_20_N_2_S_4_, the 1,3-dithiol-2-yl­idene and 4,5-diaza­fluoren-9-one (dafone) groups are almost coplanar, making a dihedral angle of only 5.65 (4)°. The two benzyl groups are on different sides of the 1,3-dithiol-2-yl­idene ring, forming a dihedral angle of 35.54 (2)°.

## Related literature

For general synthesis, see: Sako *et al.* (1996 ▶); Wong *et al.* (2005 ▶); Amriou *et al.* (2006 ▶); Baudron & Hosseini (2006 ▶). For the crystal structures of related compounds, see: Rillema *et al.* (2007 ▶); Zhang *et al.* (2003 ▶).
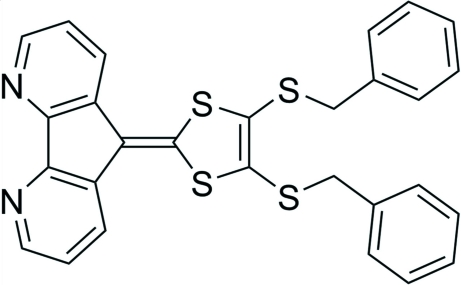


## Experimental

### 

#### Crystal data

C_28_H_20_N_2_S_4_
*M* *_r_* = 512.70
Monoclinic, 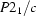

*a* = 16.5133 (4) Å
*b* = 11.4036 (3) Å
*c* = 13.1406 (3) Å
β = 100.460 (1)°
*V* = 2435.06 (10) Å^3^
*Z* = 4
Mo *K*α radiation
μ = 0.41 mm^−1^
*T* = 296 (2) K
0.30 × 0.15 × 0.15 mm

#### Data collection

Bruker SMART 1K CCD area-detector diffractometer
Absorption correction: multi-scan (*SADABS*; Bruker, 2000 ▶) *T*_min_ = 0.93, *T*_max_ = 0.95
22070 measured reflections
4701 independent reflections
3365 reflections with *I* > 2σ(*I*)
*R*_int_ = 0.028

#### Refinement

*R*[*F* ^2^ > 2σ(*F* ^2^)] = 0.039
*wR*(*F* ^2^) = 0.137
*S* = 1.00
4701 reflections
307 parameters
H-atom parameters constrained
Δρ_max_ = 0.27 e Å^−3^
Δρ_min_ = −0.26 e Å^−3^

### 

Data collection: *APEX2* (Bruker, 2004 ▶); cell refinement: *SAINT* (Bruker, 2004 ▶); data reduction: *SAINT*; program(s) used to solve structure: *SHELXS97* (Sheldrick, 2008 ▶); program(s) used to refine structure: *SHELXL97* (Sheldrick, 2008 ▶); molecular graphics: *ORTEP-3 for Windows* (Farrugia, 1997 ▶); software used to prepare material for publication: *SHELXL97* and *PLATON* (Spek, 2003 ▶).

## Supplementary Material

Crystal structure: contains datablocks I, global. DOI: 10.1107/S1600536808018667/rt2019sup1.cif

Structure factors: contains datablocks I. DOI: 10.1107/S1600536808018667/rt2019Isup2.hkl

Additional supplementary materials:  crystallographic information; 3D view; checkCIF report

## Figures and Tables

**Fig. 1.**
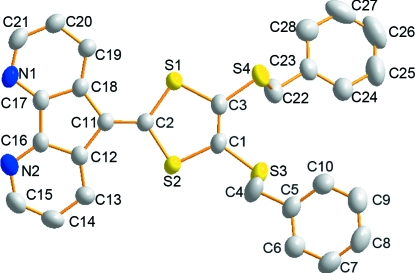
The molecular structure of the title compound, with atom labels and 30% probability displacement ellipsoids for non-H atoms. H atoms attached to carbon atoms are omitted for clarity. Symmetry codes: (i) x, y, z; (ii) -x, y + 1/2, -z + 1/2; (iii) -x, -y, -z. (iv) x, -y - 1/2, z - 1/2;

**Fig. 2.**
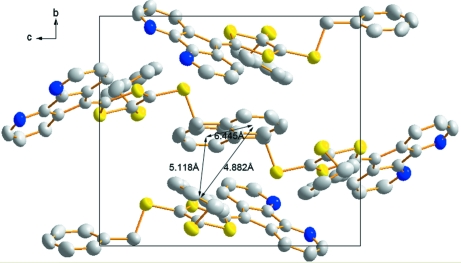
Packing diagram of the title compound. H atoms are omitted for clarity.

## supplementary crystallographic information

### Comment

The two coordinating nitrogen atoms in dafone have a larger bite distance (2.99 Å) compared to phenanthroline (2.65 Å). The larger N—N bite distance enforced by the rigid five-membered central ring leads to unequal binding by the two nitrogen atoms with small metal ions (Zhang *et al.*, 2003;). The title compound was synthesized illustrating the coordination mode of dafone.

The molecular structure of the title compound was determined by X-ray analysis (Fig. 1). The 1,3-dithiol-2-ylidene and dafone groups in the structure exhibits a near planar geometry with a dihedral angle 5.65°, and can be explained by the steric repulsion between the S of the dithiole and the *peri* H of dafone. In the experimentally determined structure, the distances between S1—H19 and S2—H13 are 2.578 and 2.597 Å respectively, although this is still less than the sum of the van der Waals radii (2.91 Å). The two benzyl group are located on different sides of the 1,3-dithiol-2-ylidene plane exhibiting a dihedral angle of 35.5° between them. The exocyclic C11=C12 bond is sightly longer [1.364 Å] than the standard olefinic (*R*_2_C=C*R*_2_) bond length of 1.33 Å and is typical for 1,3-dithiol-2-ylidene groups [average 1.36 Å].

Fig.2 shows the packing in the unit cell and two types of π-π interactions of the benzyl groups: offset face-to-face and edge-to-face. The distance *Cg*···*Cg* between the centroids of the two adjacent benzyl groups is 6.445 Å for offset face-to-face, 4.882 and 5.118 Å for edge-to-face.

### Experimental

Dafone was synthesized by the oxidation of 1,10-phenanthroline with alkaline potassium permanganate in 40.6% yields according to the procedure reported in literature (Wong *et al.*, 2005;). The cross coupling reaction of dafone with the corresponding 4,5-bis(benzylthio)-1,3-dithione- 2-thione in the presence of triethyl phosphite under a nitrogen atmosphere afforded the title compound along with the mixture of the selfcoupling product of 4,5-bis(benzylthio)-1,3-dithione-2-thione.

Crystals suitable for single-crystal X-ray diffraction were grown by recrystallisation from ethanol. ^1^H-NMR(CDCl_3_, 400 MHz) *δ*/nm: 8.689–8.700(d, 2H); 7.936–7.955(d, 2H), 7.310–7.407(m, 8H).

### Refinement

All the non-hydrogen atoms were located from the Fourier maps, and were refined anisotropically. All the H atoms were positioned geometrically, and was allowed to ride on their corresponding parent atoms with *U*_iso_ = 1.2 *U*_eq_.

### Crystal data

**Table:**

C_28_H_20_N_2_S_4_	*F*_000_ = 1064
*M**_r_* = 512.70	*D*_x_ = 1.399 Mg m^−^^3^
Monoclinic, *P*2_1_/*c*	Mo *K*α radiation λ = 0.71073 Å
Hall symbol: -P 2ybc	Cell parameters from 3616 reflections
*a* = 16.5133 (4) Å	θ = 1.3–28.1º
*b* = 11.4036 (3) Å	µ = 0.41 mm^−^^1^
*c* = 13.1406 (3) Å	*T* = 296 (2) K
β = 100.2460 (10)º	Prism, yellow
*V* = 2435.06 (10) Å^3^	0.30 × 0.15 × 0.15 mm
*Z* = 4	

### Data collection

**Table:**

Bruker SMART 1K CCD area-detector diffractometer	4701 independent reflections
Radiation source: fine-focus sealed tube	3365 reflections with *I* > 2σ(*I*)
Monochromator: graphite	*R*_int_ = 0.028
Detector resolution: 8.192 pixels mm^-1^	θ_max_ = 26.0º
*T* = 296(2) K	θ_min_ = 1.3º
Thin–slice ω scans	*h* = −20→16
Absorption correction: multi-scan(SADABS; Bruker, 2000)	*k* = −11→14
*T*_min_ = 0.93, *T*_max_ = 0.95	*l* = −16→14
22070 measured reflections	

### Refinement

**Table:**

Refinement on *F*^2^	Secondary atom site location: difference Fourier map
Least-squares matrix: full	Hydrogen site location: inferred from neighbouring sites
*R*[*F*^2^ > 2σ(*F*^2^)] = 0.039	H-atom parameters constrained
*wR*(*F*^2^) = 0.137	*w* = 1/[σ^2^(*F*_o_^2^) + (0.088*P*)^2^ + 0.1086*P*] where *P* = (*F*_o_^2^ + 2*F*_c_^2^)/3
*S* = 1.01	(Δ/σ)_max_ < 0.001
4701 reflections	Δρ_max_ = 0.27 e Å^−^^3^
307 parameters	Δρ_min_ = −0.25 e Å^−^^3^
Primary atom site location: structure-invariant direct methods	Extinction correction: none

### Fractional atomic coordinates and isotropic or equivalent isotropic displacement parameters (Å^2^)

**Table:**

	*x*	*y*	*z*	*U*_iso_*/*U*_eq_	
C1	0.26661 (13)	0.3957 (2)	0.12381 (18)	0.0443 (6)	
C2	0.11648 (12)	0.37608 (19)	0.01975 (16)	0.0388 (5)	
C3	0.22308 (13)	0.3509 (2)	0.19150 (18)	0.0437 (6)	
C4	0.40701 (15)	0.3175 (3)	0.0638 (2)	0.0651 (8)	
H4A	0.3799	0.2431	0.0702	0.078*	
H4B	0.3930	0.3432	−0.0075	0.078*	
C5	0.49893 (14)	0.3027 (2)	0.0933 (2)	0.0513 (6)	
C6	0.55077 (16)	0.3460 (2)	0.0315 (2)	0.0580 (7)	
H6	0.5292	0.3858	−0.0290	0.070*	
C7	0.63543 (16)	0.3306 (2)	0.0590 (2)	0.0651 (8)	
H7	0.6702	0.3599	0.0167	0.078*	
C8	0.66740 (16)	0.2728 (3)	0.1474 (2)	0.0660 (8)	
H8	0.7240	0.2628	0.1657	0.079*	
C9	0.61659 (17)	0.2296 (2)	0.2091 (2)	0.0652 (8)	
H9	0.6387	0.1897	0.2694	0.078*	
C10	0.53260 (16)	0.2442 (2)	0.1833 (2)	0.0595 (7)	
H10	0.4985	0.2148	0.2263	0.071*	
C11	0.04782 (12)	0.37793 (19)	−0.05529 (17)	0.0389 (5)	
C12	0.03983 (14)	0.42998 (19)	−0.15886 (17)	0.0415 (5)	
C13	0.09229 (15)	0.4942 (2)	−0.20960 (19)	0.0532 (6)	
H13	0.1463	0.5095	−0.1782	0.064*	
C14	0.06161 (17)	0.5345 (2)	−0.3080 (2)	0.0619 (7)	
H14	0.0952	0.5770	−0.3444	0.074*	
C15	−0.01918 (17)	0.5114 (2)	−0.35224 (19)	0.0592 (7)	
H15	−0.0383	0.5402	−0.4184	0.071*	
C16	−0.04129 (13)	0.41232 (19)	−0.21187 (17)	0.0420 (5)	
C17	−0.08682 (13)	0.34911 (19)	−0.14401 (17)	0.0426 (6)	
C18	−0.03350 (12)	0.32953 (19)	−0.04860 (17)	0.0389 (5)	
C19	−0.06498 (13)	0.2702 (2)	0.02749 (18)	0.0454 (6)	
H19	−0.0327	0.2552	0.0917	0.054*	
C20	−0.14634 (14)	0.2337 (2)	0.0048 (2)	0.0522 (6)	
H20	−0.1696	0.1938	0.0543	0.063*	
C21	−0.19258 (14)	0.2566 (2)	−0.0910 (2)	0.0554 (7)	
H21	−0.2467	0.2299	−0.1041	0.067*	
C22	0.27286 (17)	0.4698 (2)	0.37016 (19)	0.0585 (7)	
H22A	0.3122	0.5104	0.3361	0.070*	
H22B	0.2207	0.5109	0.3541	0.070*	
C23	0.30247 (15)	0.4705 (2)	0.48452 (19)	0.0487 (6)	
C24	0.38114 (16)	0.5099 (2)	0.5233 (2)	0.0605 (7)	
H24	0.4152	0.5329	0.4777	0.073*	
C25	0.4097 (2)	0.5156 (3)	0.6274 (3)	0.0802 (9)	
H25	0.4626	0.5431	0.6518	0.096*	
C26	0.3614 (3)	0.4814 (3)	0.6953 (3)	0.0880 (11)	
H26	0.3813	0.4845	0.7661	0.106*	
C27	0.2830 (3)	0.4420 (3)	0.6591 (3)	0.0821 (10)	
H27	0.2497	0.4193	0.7057	0.099*	
C28	0.25280 (17)	0.4359 (2)	0.5533 (2)	0.0623 (7)	
H28	0.1997	0.4087	0.5292	0.075*	
N1	−0.16515 (11)	0.31450 (18)	−0.16673 (16)	0.0504 (5)	
N2	−0.07133 (12)	0.45076 (19)	−0.30653 (15)	0.0525 (5)	
S1	0.11858 (3)	0.32501 (5)	0.14525 (4)	0.0451 (2)	
S2	0.21191 (3)	0.42865 (6)	0.00076 (5)	0.0470 (2)	
S3	0.25999 (4)	0.31922 (6)	0.32190 (5)	0.0541 (2)	
S4	0.37216 (4)	0.42555 (6)	0.14861 (5)	0.0535 (2)	

### Atomic displacement parameters (Å^2^)

**Table:**

	*U*^11^	*U*^22^	*U*^33^	*U*^12^	*U*^13^	*U*^23^
C1	0.0350 (12)	0.0470 (14)	0.0480 (14)	−0.0001 (10)	−0.0005 (11)	−0.0063 (11)
C2	0.0353 (11)	0.0382 (13)	0.0419 (13)	−0.0015 (10)	0.0045 (10)	−0.0024 (10)
C3	0.0407 (12)	0.0413 (13)	0.0454 (14)	0.0020 (10)	−0.0022 (11)	−0.0010 (10)
C4	0.0392 (13)	0.084 (2)	0.0697 (18)	−0.0049 (13)	0.0033 (12)	−0.0222 (15)
C5	0.0391 (12)	0.0519 (15)	0.0618 (17)	−0.0012 (11)	0.0058 (12)	−0.0124 (13)
C6	0.0521 (15)	0.0583 (17)	0.0635 (18)	0.0064 (12)	0.0102 (13)	−0.0009 (13)
C7	0.0462 (15)	0.0671 (19)	0.085 (2)	−0.0009 (13)	0.0201 (15)	−0.0047 (16)
C8	0.0417 (14)	0.0595 (18)	0.093 (2)	0.0095 (13)	0.0005 (15)	−0.0164 (16)
C9	0.0637 (18)	0.0517 (17)	0.0730 (19)	0.0056 (14)	−0.0077 (16)	−0.0025 (14)
C10	0.0593 (17)	0.0562 (17)	0.0620 (18)	−0.0127 (13)	0.0080 (14)	−0.0018 (13)
C11	0.0344 (11)	0.0399 (13)	0.0415 (13)	0.0008 (10)	0.0045 (10)	−0.0010 (10)
C12	0.0409 (12)	0.0427 (13)	0.0399 (13)	0.0018 (10)	0.0043 (10)	−0.0025 (10)
C13	0.0505 (14)	0.0589 (17)	0.0499 (15)	−0.0058 (12)	0.0081 (12)	0.0065 (12)
C14	0.0694 (18)	0.0680 (18)	0.0508 (16)	−0.0089 (14)	0.0175 (14)	0.0094 (14)
C15	0.0690 (17)	0.0673 (18)	0.0394 (15)	0.0036 (14)	0.0048 (13)	0.0064 (13)
C16	0.0389 (12)	0.0458 (14)	0.0393 (13)	0.0022 (10)	0.0019 (10)	−0.0053 (11)
C17	0.0379 (12)	0.0436 (14)	0.0437 (14)	0.0043 (10)	0.0007 (10)	−0.0051 (11)
C18	0.0330 (11)	0.0402 (13)	0.0427 (13)	0.0019 (9)	0.0043 (10)	−0.0050 (10)
C19	0.0391 (13)	0.0488 (14)	0.0479 (14)	−0.0030 (10)	0.0070 (11)	−0.0007 (11)
C20	0.0398 (13)	0.0568 (16)	0.0609 (17)	−0.0034 (11)	0.0114 (12)	0.0008 (13)
C21	0.0341 (13)	0.0568 (16)	0.0738 (19)	−0.0031 (11)	0.0055 (13)	−0.0037 (14)
C22	0.0678 (17)	0.0483 (15)	0.0526 (16)	0.0021 (13)	−0.0080 (13)	−0.0018 (12)
C23	0.0520 (14)	0.0426 (14)	0.0479 (15)	0.0089 (11)	−0.0010 (12)	−0.0001 (11)
C24	0.0580 (16)	0.0592 (17)	0.0609 (18)	−0.0002 (13)	0.0014 (14)	0.0011 (13)
C25	0.085 (2)	0.069 (2)	0.072 (2)	0.0017 (17)	−0.0248 (18)	−0.0088 (17)
C26	0.135 (3)	0.068 (2)	0.051 (2)	0.028 (2)	−0.011 (2)	−0.0131 (16)
C27	0.129 (3)	0.064 (2)	0.061 (2)	0.030 (2)	0.041 (2)	0.0126 (16)
C28	0.0613 (17)	0.0548 (17)	0.072 (2)	0.0086 (13)	0.0143 (15)	0.0028 (14)
N1	0.0345 (10)	0.0572 (13)	0.0560 (13)	−0.0001 (9)	−0.0013 (9)	−0.0045 (10)
N2	0.0558 (13)	0.0602 (14)	0.0388 (12)	0.0043 (10)	0.0011 (10)	−0.0009 (10)
S1	0.0392 (3)	0.0531 (4)	0.0412 (4)	−0.0035 (3)	0.0020 (3)	0.0036 (3)
S2	0.0357 (3)	0.0598 (4)	0.0443 (4)	−0.0074 (3)	0.0040 (3)	0.0001 (3)
S3	0.0621 (4)	0.0465 (4)	0.0470 (4)	−0.0019 (3)	−0.0083 (3)	0.0044 (3)
S4	0.0353 (3)	0.0593 (4)	0.0626 (5)	−0.0048 (3)	−0.0006 (3)	−0.0127 (3)

### Geometric parameters (Å, °)

**Table:**

C1—C3	1.340 (3)	C14—H14	0.9300
C1—S2	1.747 (2)	C15—N2	1.328 (3)
C1—S4	1.749 (2)	C15—H15	0.9300
C2—C11	1.364 (3)	C16—N2	1.329 (3)
C2—S1	1.744 (2)	C16—C17	1.455 (3)
C2—S2	1.745 (2)	C17—N1	1.334 (3)
C3—S1	1.750 (2)	C17—C18	1.416 (3)
C3—S3	1.751 (2)	C18—C19	1.383 (3)
C4—C5	1.508 (3)	C19—C20	1.387 (3)
C4—S4	1.822 (3)	C19—H19	0.9300
C4—H4A	0.9700	C20—C21	1.377 (3)
C4—H4B	0.9700	C20—H20	0.9300
C5—C6	1.372 (3)	C21—N1	1.338 (3)
C5—C10	1.385 (3)	C21—H21	0.9300
C6—C7	1.391 (3)	C22—C23	1.495 (3)
C6—H6	0.9300	C22—S3	1.829 (2)
C7—C8	1.358 (4)	C22—H22A	0.9700
C7—H7	0.9300	C22—H22B	0.9700
C8—C9	1.359 (4)	C23—C28	1.382 (3)
C8—H8	0.9300	C23—C24	1.383 (3)
C9—C10	1.378 (4)	C24—C25	1.367 (4)
C9—H9	0.9300	C24—H24	0.9300
C10—H10	0.9300	C25—C26	1.356 (5)
C11—C18	1.469 (3)	C25—H25	0.9300
C11—C12	1.469 (3)	C26—C27	1.374 (5)
C12—C13	1.392 (3)	C26—H26	0.9300
C12—C16	1.410 (3)	C27—C28	1.392 (4)
C13—C14	1.381 (3)	C27—H27	0.9300
C13—H13	0.9300	C28—H28	0.9300
C14—C15	1.382 (4)		
			
C3—C1—S2	116.50 (17)	N2—C16—C12	125.4 (2)
C3—C1—S4	125.80 (19)	N2—C16—C17	126.1 (2)
S2—C1—S4	117.69 (14)	C12—C16—C17	108.48 (19)
C11—C2—S1	124.27 (16)	N1—C17—C18	125.2 (2)
C11—C2—S2	123.16 (17)	N1—C17—C16	126.5 (2)
S1—C2—S2	112.55 (12)	C18—C17—C16	108.24 (19)
C1—C3—S1	116.50 (18)	C19—C18—C17	117.57 (19)
C1—C3—S3	126.42 (18)	C19—C18—C11	133.8 (2)
S1—C3—S3	117.07 (14)	C17—C18—C11	108.60 (19)
C5—C4—S4	109.37 (17)	C18—C19—C20	117.8 (2)
C5—C4—H4A	109.8	C18—C19—H19	121.1
S4—C4—H4A	109.8	C20—C19—H19	121.1
C5—C4—H4B	109.8	C21—C20—C19	119.9 (2)
S4—C4—H4B	109.8	C21—C20—H20	120.1
H4A—C4—H4B	108.2	C19—C20—H20	120.1
C6—C5—C10	118.7 (2)	N1—C21—C20	124.6 (2)
C6—C5—C4	120.9 (2)	N1—C21—H21	117.7
C10—C5—C4	120.4 (2)	C20—C21—H21	117.7
C5—C6—C7	120.3 (3)	C23—C22—S3	110.54 (17)
C5—C6—H6	119.9	C23—C22—H22A	109.5
C7—C6—H6	119.9	S3—C22—H22A	109.5
C8—C7—C6	120.2 (3)	C23—C22—H22B	109.5
C8—C7—H7	119.9	S3—C22—H22B	109.5
C6—C7—H7	119.9	H22A—C22—H22B	108.1
C7—C8—C9	119.9 (3)	C28—C23—C24	118.7 (2)
C7—C8—H8	120.0	C28—C23—C22	122.0 (2)
C9—C8—H8	120.0	C24—C23—C22	119.3 (2)
C8—C9—C10	120.7 (3)	C25—C24—C23	121.2 (3)
C8—C9—H9	119.7	C25—C24—H24	119.4
C10—C9—H9	119.7	C23—C24—H24	119.4
C9—C10—C5	120.2 (3)	C26—C25—C24	120.4 (3)
C9—C10—H10	119.9	C26—C25—H25	119.8
C5—C10—H10	119.9	C24—C25—H25	119.8
C2—C11—C18	127.0 (2)	C25—C26—C27	119.6 (3)
C2—C11—C12	127.01 (19)	C25—C26—H26	120.2
C18—C11—C12	105.95 (18)	C27—C26—H26	120.2
C13—C12—C16	117.1 (2)	C26—C27—C28	120.7 (3)
C13—C12—C11	134.1 (2)	C26—C27—H27	119.6
C16—C12—C11	108.7 (2)	C28—C27—H27	119.6
C14—C13—C12	117.9 (2)	C23—C28—C27	119.3 (3)
C14—C13—H13	121.1	C23—C28—H28	120.3
C12—C13—H13	121.1	C27—C28—H28	120.3
C13—C14—C15	119.7 (2)	C17—N1—C21	115.0 (2)
C13—C14—H14	120.1	C15—N2—C16	115.5 (2)
C15—C14—H14	120.1	C2—S1—C3	97.15 (10)
N2—C15—C14	124.3 (2)	C2—S2—C1	97.19 (11)
N2—C15—H15	117.8	C3—S3—C22	98.28 (11)
C14—C15—H15	117.8	C1—S4—C4	99.62 (11)
